# The Spitting Nuisance

**Published:** 1897-04

**Authors:** 


### The Spitting Nuisance.

The shadow of an approaching reform seems to be near us. We must not be too hopeful, for the movement has scarcely begun, and it is impossible to say what serious obstacles it may meet. There is, however, a slow awakening on the part of the community to the knowledge that miscellaneous spitting is a dangerous practice. It argues ill for human nature, that so little was accomplished in restraining this habit as long as it was considered merely as disgusting. Rules in regard to it were liberally posted in conveyances and public places, yet were but little obeyed. Now that it has been proved that disease may be and is conveyed by this method, the regulations in regard to it pass out of the domain of mere discipline or manners and become part of hygiene. Accordingly we find that boards of health are moving in the matter, and we may hope that a public sentiment will steadily grow in favor of the strictest prohibition of the habit.

Looking at the matter practically, it seems that a large proportion of spitting is merely an imitative habit. As far as regards the tobacco-chewers, we may place them within the pale of those who have no rights that anybody is bound to respect. The right of any man to make a hog of himself may be allowed as long as his hoggishness affects only himself and those who willingly associate with him. In conveyances and public places generally, the tobacco-chewer thrusts himself and his habit upon every one, and there has seemed no limit to his offensiveness.

The habit is never excusable, but is perhaps more flagrant in certain college circles than anywhere else. In medical and dental schools, for instance, there is a fine field for beginning reform. Most of the pupils are young men to whom tobacco in any form is injurious; the buildings are the property of the college, and the officers are considered to have some features of parental authority. Why is it, then, that students are permitted to indulge in habits which cause an hour's stay of a class to give to a lecture-room the aspect of a stable? Why should not the college authorities make rules for the protection of their property and in the interest of cleanliness and health? Certainly it is not because the habit is agreeable or believed to be harmless. It is simply because there is a fear that some students would take offense and the college would lose a little in fees.

A systematic effort to suppress spitting in the street-cars has been begun lately in Philadelphia. For years the cars have contained a notice, posted by the company, declaring, with that indifference to correct English that seems to be constant with street-car companies, that "spitting on the floor of this car is prohibited;" but very little attention was paid to it, and so the practice was not really prohibited. Lately the Board of Health prepared and posted in each car a notice to the effect that spitting on the floor is forbidden. Observation seems to show that a marked improvement has taken place. The car-floors seem to be cleaner, and "for this relief, much thanks." Whether the reform will be permanent can not now be predicted. It is not clear what steps will be taken to prevent violation of the rules. The Board has rather summary process for the interdiction of any practice or the removal of any nuisance, but issue in this case is unusual. It does not seem possible that passengers who persistently spit on the car-floor can be either ejected or prohibited from riding. Where there is a will, there is, or soon will be, a way, and the regulation will work some change for the better. Meanwhile, we hope some missionary work may be entered upon, some employment of moral suasion, enforcement of mild discipline and display of good example. We suggest that those in charge of schools and colleges begin to draw the lines of student behavior more strictly and try to abolish altogether the practice of chewing tobacco.

Tobacco-chewers are, however, not the only persistent and disgusting spitters. Post-nasal catarrhs are very common and give rise to the habit. Some people, as noted above, are spitters merely from habit; others, especially half-grown boys, possibly indulge in the practice because it is supposed to be "manly."

The main danger is, of course, from the consumptive, but it is probable that without that danger but little reform would be accomplished, for it is largely because of the liability to communicate disease that the forbidding of the general practice of spitting has been taken up by the sanitary authorities.—*Dietetic and Hygienic Gazette.*

